# Isolation and Characterization of Fungal Endophytes Isolated from Medicinal Plant *Ephedra pachyclada* as Plant Growth-Promoting

**DOI:** 10.3390/biom11020140

**Published:** 2021-01-22

**Authors:** Ahmed Mohamed Aly Khalil, Saad El-Din Hassan, Sultan M. Alsharif, Ahmed M. Eid, Emad El-Din Ewais, Ehab Azab, Adil A. Gobouri, Amr Elkelish, Amr Fouda

**Affiliations:** 1 Department of Botany and Microbiology, Faculty of Science, Al-Azhar University, Nasr City, Cairo 11884, Egypt; khalilahmed_1980@hotmail.com (A.M.A.K.); aeidmicrobiology@azhar.edu.eg (A.M.E.); emad_ewais@azhar.edu.eg (E.E.-D.E.); 2 Biology Department, College of Science, Taibah University, Yanbu 41911, Saudi Arabia; 3 Biology Department, Faculty of Science, Taibah University, Al Madinah P.O. Box 887, Saudi Arabia; Ssharif@taibahu.edu.sa; 4 Department of Biotechnology, College of Science, Taif University, P.O. Box 11099, Taif 21944, Saudi Arabia; e.azab@tu.edu.sa; 5 Botany and Microbiology Department, Faculty of Science, Zagazig University, Zagazig 44519, Sharkia, Egypt; 6 Department of Chemistry, College of Science, Taif University, P.O. Box 11099, Taif 21944, Saudi Arabia; a.gobouri@tu.edu.sa; 7 Botany Department, Faculty of Science, Suez Canal University, Ismailia 41522, Egypt; amr.elkelish@science.suez.edu.eg

**Keywords:** Endophytic fungi, *Ephedra pachyclada*, Ascomycota, plant growth-promoting, bioactive metabolites, *Zea mays* L.

## Abstract

Endophytic fungi are widely present in internal plant tissues and provide different benefits to their host. Medicinal plants have unexplored diversity of functional fungal association; therefore, this study aimed to isolate endophytic fungi associated with leaves of medicinal plants *Ephedra pachyclada* and evaluate their plant growth-promoting properties. Fifteen isolated fungal endophytes belonging to Ascomycota, with three different genera, *Penicillium, Alternaria,* and *Aspergillus*, were obtained from healthy leaves of *E. pachyclada*. These fungal endophytes have varied antimicrobial activity against human pathogenic microbes and produce ammonia and indole acetic acid (IAA), in addition to their enzymatic activity. The results showed that *Penicillium commune* EP-5 had a maximum IAA productivity of 192.1 ± 4.04 µg mL^−1^ in the presence of 5 µg mL^−1^ tryptophan. The fungal isolates of *Penicillium crustosum* EP-2*, Penicillium chrysogenum* EP-3, and *Aspergillus flavus* EP-14 exhibited variable efficiency for solubilizing phosphate salts. Five representative fungal endophytes of *Penicillium crustosum* EP-2, *Penicillium commune* EP-5, *Penicillium caseifulvum* EP-11, *Alternaria tenuissima* EP-13, and *Aspergillus flavus* EP-14 and their consortium were selected and applied as bioinoculant to maize plants. The results showed that *Penicillium commune* EP-5 increased root lengths from 15.8 ± 0.8 to 22.1 ± 0.6. Moreover, the vegetative growth features of inoculated maize plants improved more than the uninoculated ones.

## 1. Introduction

The improvement of food sources and crops are receiving more attention in recent years due to high population growth; subsequently, demand for natural products for food, medicine, energy, and other biotechnological aspects is increasing [1]. Excessive use of chemical fertilizers has several environmental hazards, such as increased soil pollution, reduced microbial diversity in soil, decreased food safety, and the leaching of minerals into the groundwater [2,3]. Recently, natural agricultural strategies are urgently required to replace the long-term application of chemical fertilizers to enhance crop productivity and reduce environmental pollutions that arise from chemical compounds [4,5]. Fungal endophytes are those colonized intra- or intercellular spaces of plant tissues that do not show any illness symptoms [6]. Fungal endophytes have various mechanisms to promote plant growth, including phytostimulation, biocontrol, and biofertilizations [7,8]; for example, plant growth-promoting fungi (PGPF) directly promote plant growth by the production of phytohormones such as indole-3-acetic acid (IAA) and gibberellic acid [9]. Fungal IAA synergistically reacts with endogenous plant IAA, which then stimulate plant growth. In the same regard, PGPF promotes plant growth by nitrogen fixation and phosphate solubilizations [10], the production of different enzymes, such as amylase, cellulase, urease, catalase, and protease [7,11], in addition to ammonia production [12]. Moreover, fungal endophytes have high potential to protect plants against different pathogens and, hence, reduce crop loss through the secretion of different bioactive compounds such as antibiotics [13].

The Sinai Peninsula represents 6% of the Egyptian land area and is characterized by extreme climates, i.e., an arid to semiarid environment, moderate temperature, and winter precipitation. The Saint Katherine Protectorate is situated in the upper Sinai massif and extends over 4350 km² of South Sinai, making it the fourth-largest protectorate in Egypt [14]. Saint Katherine Protectorate contains a high diversity of medicinal plants that harbor different endophytic microorganisms with unique metabolites of agricultural and biotechnological importance [15]. To date, few studies have discussed the potentiality of fungal endophytes isolated from the medicinal plants of Sinai as plant growth-promoting microorganisms.

*Ephedra* is represented as the only genus of the *Ephedraceae* family which is distributed worldwide. *Ephedra* spp. have high efficiency to adapt to climate change, especially in arid and semi-arid habitats [16]. Therefore, this species harbors various microbial endophytes that increase plant tolerance to biotic and abiotic stress. Out of 132 microbial endophytes obtained from 18 medicinal plants collected from Saint Katherine Protectorate, *Ephedra alata* is the second plant in association with 12 bacterial and fungal endophytes that possess antimicrobial and antiviral activity [17,18]. In the same manner, the highest fungal endophytic diversity was shown in *Ephedra nebrodensis* and *Rosmarinus officinalis* [19]. However, no studies have been conducted to evaluate the potential plant growth-promoting bioactivity of endophytic fungi obtained from *Ephedra* spp.

Therefore, this study focused on isolation and identification of culturable fungal endophytes from *E. pachyclada* which is a domestic inhabitant in the extremely harsh climate of Saint Katherine Protectorate. Plant growth-promoting activities, including extracellular enzymatic production (amylase, carboxymethyl cellulase, gelatinase, pectinase, xylanase, and catalase), antimicrobial activity against pathogenic Gram-positive, Gram-negative and unicellular fungi, inorganic phosphate solubilization, ammonia production, and qualitative and quantitative IAA productions were investigated. Afterward, the potential application of representative fungal endophytes as bioinculants for improving the performance growth of *Zea mays* L. was evaluated.

## 2. Materials and Methods

### 2.1. Material Used

All chemicals used to prepare different reagents used in this study as well as the medium components were obtained from Sigma Aldrich, Cairo, Egypt. All reactions were achieved using distilled water (dis. H_2_O). The internal transcribed spacer (ITS) Kits and universal primers for molecular identification were obtained from QIAGEN.

### 2.2. Plant Sampling and Study Area

*Ephedra pachyclada* Boiss plants (family *Ephedraceae*) were collected from Wadi Selebat (lat 28.545493 to 28.543339 N, long 33.933707 to 33.932984 E), the World Heritage site of Saint Katherine (WHS No. 954), South Sinai, Egypt. Samples of healthy plants were put in sterile Zipper bags and imparted to the laboratory in portable cool chambers (4 °C). The plant identification has been achieved at the herbarium of Botany and Microbiology Department, Faculty of Science, Al-Azhar University with the help of Dr. AlBaraa S. M. ElSaied, (associate professor of Plant Ecology at the same Department, who is one of the herbarium experts).

### 2.3. Isolation of Fungal Endophytes

The plant leaves and stems were washed with running tap water; leaf segments were equally cut by sterilized scalpel from the mid portions of healthy leaves to include the midrib. The cut segments were surface sterilized by immersing into the following series of solutions: sterile dis. H_2_O for 60 s, 70% ethanol for 60 s, 2.5% sodium hypochlorite for 4 min, 70% ethanol for 30 s, and a final rinsing in sterile dis. H_2_O three times. About 100 µL of the final rinse water was inoculated on Malt Extract Agar (MEA) medium (Oxoid, UK) to success the surface sterilization.

The sterilized plant leaves were cut into five segments (5 mm), and 20 leaf segments per individual plant were placed on the surface of MEA plate (five segment for each plate), supplemented with 0.05 g of streptomycin sulfate per 100 mL of medium to inhibit bacterial growth and incubated at 28 °C ± 2 °C. The plates were checked daily for any fungal growth; single isolates grown out from the tissues were re-inoculated on fresh MEA plates and maintained at 4 °C in MEA slants [20].

### 2.4. Identification of Fungal Endophytes

Endophytic fungal isolates were identified based on routine cultural and morphological characteristics and microscopical features according to [21,22]. For molecular identification, genomic DNA was extracted according to the method of [23]. Fungal ITS rDNA rejoins was amplified by the primers of ITS1 (5-CTTGGTCATTTAGAGGAAGTAA-3) and ITS4 (5-TCCTCCGCTTATT GATATGC-3) [24]. The PCR mixture contained: 1 × PCR buffer, 0.5 mM MgCl_2_, 2.5 U Taq DNA polymerase (QIAGEN, Germantown, MD 20874, USA), 0.25 mM dNTP, 0.5 µM of each primer, and nearly 5 ng of the extracted genomic DNA. The PCR was performed in a DNA Engine Thermal Cycler (PTC-200, BIO-RAD, USA) with 94 °C for 3 min, followed by 30 cycles of 94 °C for 30 s, 55 °C for 30 s, and 72 °C for 1 min, followed by final extension performed at 72 °C for 10 min. The PCR products were checked for the expected size on 1% agarose gel and were commercially sequenced on GATC Biotech. company using ABI 3730xl DNA sequencers through partner Sigma Aldrich, Cairo, Egypt. The fungal ITS sequences at this study have been deposited in GenBank under accession numbers MN954764–MN954778. The sequences were compared against the GenBank database using the ClustalX 1.8 software package (http://www.clustal.org/clustal2) [25]. Phylogenetic analysis was constructed by the neighbor-joining method using MEGA v6.1 software, with confidence tested by bootstrap analysis (1000 repeats).

### 2.5. Investigation of the Plant Growth-Promoting Traits

#### 2.5.1. Detection of Fungal Endophytes Extracellular Enzymatic Activity

Endophytic fungal enzyme production was qualitatively determined by the agar plate-based method. Firstly, endophytic fungal strains were grown on Yeast-malt extract (YM) agar medium which contains yeast extract 3 g L^−1^; malt extract 3 g L^−1^; glucose 10 g L^−1^; peptone 5 g L^−1^; agar 15 g L^−1^; 1 L d.H_2_O; pH 6.7 at 28 °C for 7 days. After the incubation period, 5 mm mycelial plugs were cut and individually placed on minimal agar medium contains NaNO_3,_ 6 g L^−1^, KCl, 5 g L^−1^; KH_2_PO_4_, 1.5 g L^−1^; MgSO_4_·7H_2_O, 0.5 g L^−1^; ZnSO_4_, 0.01 g L^−1^; FeSO_4_, 0.01 g L^−1^ and agar, 15 g L^−1^; pH 5.0 supplemented with 1% *w*/*v* of one of the following substrates (soluble starch, carboxymethyl cellulose (CMC), gelatin, pectin, and xylan) for detecting amylase, carboxymethyl cellulase (CMCase), gelatinase, pectinase and xylanase activities, respectively.

Plates were incubated at 28 °C for 7 days, effectiveness detected by flooding plates with 1% iodine for amylase and CMCase activity [26,27]. Acidic mercuric chloride was used to visualize gelatinase activity, pectinase activity uncovered by 5 N HCl, while xylan biodegradation was assessed by absolute ethyl alcohol. The functional role of exoenzymes was indicated by measuring (mm) the halo zones surrounding the fungal colony.

For catalase activity, a drop of hydrogen peroxide (3%) was added to an endophytic isolated colony and observed for the formation of oxygen. Vigorous bubbling indicates a strong catalase reaction [28].

#### 2.5.2. Antimicrobial Activity of Fungal Endophytes

Endophytic fungal strains were grown on potato dextrose (PD) agar medium for 7 days at 28 °C. After that, five discs (5 mm) of each isolate were picked and inoculated individually in 500 mL Erlenmeyer flasks containing 200 mL of PD broth and incubated for 15 days under mild shaking (150 rpm) at 25 ± 2 °C in dark conditions.

Antimicrobial activity of fungal endophytes was assayed against Gram-positive bacteria (*Staphylococcus aureus*, ATCC 6538 and *Bacillus subtilis*, ATCC 6633), Gram-negative bacteria (*Escherichia coli*, ATCC 8739; *Salmonella typhimurium* ATCC 14028 and *Pseudomonas aeruginosa*, ATCC 9027), and unicellular fungi (*Candida albicans*, ATCC 10231). These strains were obtained from the “Physiology and Classification of Microorganisms Lab”, Faculty of Science, Al-Azhar University, Cairo, Egypt.

The fermentative broth media was filtered and centrifuged at 10,000 rpm for 5 min to remove any cell debris and to obtain cell-free supernatant (CFS). The CFS was extracted thrice with an equal volume of ethyl acetate (100 mL of CFS mixed with 100 mL of ethyl acetate), the upper layers (ethyl acetate with metabolites) were collected, and then evaporate using a vacuumed rotary evaporator (Heidolph Hei-VAP Precision motor-lift) at 40 °C. After disposal of the solvent, 1 mg of the crude extract was dissolved in 1 mL of dimethyl sulphoxide (DMSO) and stored at 4 °C.

The preliminary antimicrobial screening was done based on the agar diffusion method [29,30]. Tested Gram-positive and Gram-negative bacteria and unicellular fungi were seeded on Muller-Hinton agar plate (Sigma-Aldrich). On each plate, three wells were cut using a sterile cork-borer (0.8 mm); every well was filled with 40 µL of crude extract, while a fourth well was filled with DMSO as a negative control. The plates were kept for 2 h at 4 °C to permit diffusion of bioactive secondary metabolites, then incubated at 35 °C for 24 h. After the incubation period, antimicrobial activity was assessed by measuring the inhibition diameter (mm) zones (IDZ). The experiment was performed in triplicates.

#### 2.5.3. Phosphate Solubilization

Inorganic phosphate solubilizing potential of endophytic fungal isolates was evaluated in vitro according to [31]. Briefly, Pikovskaya medium (containing, glucose 10 gL^−1^; Ca_3_(PO_4_)_2_ 5 g L^−1^; (NH_4_)_2_SO_4_ 0.5 g L^−1^; NaCl 0.2 g L^−1^; MgSO_4_·7H_2_O 0.1 g L^−1^; KCl 0.2 g L^−1^; FeSO_4_·7H_2_O 0.002 g L^−1^; yeast extract 0.5 g L^−1^; MnSO_4_·2H_2_O 0.002 g L^−1^; agar 15 g L^−1^; 1 L d.H_2_O; pH 6.8) was prepared. The fungal plugs were inoculated on the plates of Pikovskaya medium and incubated at 28 °C for 72 h. The diameter (mm) of clear zones around fungal plugs were measured for qualitative determination of phosphate solubilizing capacity.

#### 2.5.4. Ammonia Production

The capacity for ammonia production was assessed by inoculating endophytic fungal strains in peptone water with the composition of g L^−1^; peptone 10; NaCl 5; 1.0 L d.H_2_O, incubation for 5 days at 28 °C with 140 rpm shaking condition. After the incubation period, 1 mL Nessler’s reagent (K_2_HgI_4_ and NaOH or KOH) was added to 0.2 mL of culture supernatant, the color shift from brown to yellow is considered as the endpoint for ammonia production and increasing yellow color degree indicates the varying abilities of endophytes to producing ammonia. Control is represented by peptone water media without fungal inoculations [32,33].

#### 2.5.5. Screening and Quantification of Indole-3-Acetic Acid (IAA)

The potency of culturable endophytic fungi as IAA producer was determined according to the method described by [34] with minor modifications. Endophytic fungal strains were inoculated on Czapek Dox (CD) agar medium for 7 days at 28 °C, one disc (5 mm) of each fungal culture was inoculated in 20 mL of CD broth supplemented with 0, 1, 2, and 5 mg mL^−1^ of tryptophan and incubated at 28 °C for 14 days. Non-inoculated media were considered as controls. After incubation, 5 mL of each culture was centrifuged at 6000 rpm for 30 min, 1 mL of the supernatant was mixed with 1 drop of orthophosphoric acid, and 2 mL of Salkowski’s reagent was added (300 mL concentrated sulfuric acid, 500 mL distilled water, 15 mL 0.5 M FeCl_3_). The emergence of a pink color indicated IAA production; OD was measured at 530 nm (Jenway 6305 UV spectrophotometer). The amount of produced IAA was determined by comparing it with a standard IAA graph.

The most potent five isolates with high qualitative IAA productions were selected for further experiments to quantify the amount of IAA produced. One fungal disc of each isolate was inoculated in 20 mL of CD broth supplemented with 5 mg mL^−1^ tryptophan and incubated at 28 °C for 14 days. One mL of each culture was collected every two days from the 2nd day until the 14th day. Samples were centrifuged at 6000 rpm for 30 min and IAA production was determined as mentioned above. All IAA determination and measurements were done in triplicates.

### 2.6. Effect of the Selected Fungal Strains as Bio-Inoculant

#### 2.6.1. Effect of Endophytic Fungal Inoculation on Root Growth

The selected endophytic fungal strains (*Penicillium crustosum* EP-2, *Penicillium commune* EP-5, *Penicillium caseifulvum* EP-11, *Alternaria tenuissima* EP-13, and *Aspergillus flavus* EP-14) were used as bio-inoculant to assess their impact on the root growth of the maize plant (*Zea mays* L., Giza 9 Cultivar), which was measured as the root length (cm). Briefly, the surface of maize seeds was sterilized by immersed in 2.5% sodium hypochlorite for 4 min, then the seeds were thrice washed with sterilized distilled water. The sterilized seeds were soaked in 50 mL of CD broth media inoculated with fungal strains for 24 h. The soaked seeds were transferred to a sterilized cup containing wet sterilized filter paper and incubated for 7 days at room temperature in dark conditions. The CD broth without fungal inoculations was used as a control.

#### 2.6.2. Greenhouse Experiment

##### Experimental Design

To assess the ability of isolated fungal endophytes to promote plant growth, a pot experiment was conducted under greenhouse conditions in which plants were cultivated for 30 days at 25–30 °C and a photoperiod of 14:10 h light:dark. The experiment was designed in a completely random manner using five individual fungal isolates (EP-2, EP-5, EP-11, EP-13, and EP-14), as well as a consortium containing the five isolates (Mix_F) and five replicates for each treatment. These five fungal strains were selected based on their high activities in plant-growth-promoting activities including enzymatic and antimicrobial activities, phosphate solubilization, ammonia production, and IAA production. Non-inoculated plants are running parallel with the experiment as a control.

##### Culture Conditions

A loamy soil was collected from the top of the surface layer from an agricultural field in Menoufia governorate, Egypt (30°38′20.1′′ N 30°56′59.3′′ E). The physical characteristics of loamy sand soil, including sand, silt, and clay, was 76.8:10.9:12.2%, respectively; while chemical characteristics including P, K, Na, Ca, and Cl were 24.0, 14.0, 186.5, 27.0, and 134.0 mg Kg^−1^, respectively. The soil was air-dried, grounded, and sieved with a 2 mm sieve, mixed with quartz sand at a soil: sand ratio of 3:1, and autoclaved twice at 121 °C for one hour. Seven inoculation treatments, consisting of the five most potent fungal isolates, fungal consortium (five fungal isolates mixed), and the broth media without inoculation, which was considered as control were conducted. Two discs (5 mm) of each fungal endophyte were inoculated separately in 250 mL of CD broth. All flasks were incubated at 25 ± 2 °C for 5 days on a shaker at 180 rpm. The sterilized maize seeds were divided into seven groups, with one group for each of the five fungal strains, one group for the endophytic fungal mix (Mix_F), and one group for the control (cultivated media without fungal inoculations). The sterilized seeds could germinate under dark conditions at 25 °C; once the seed coat ruptured and the radical emerged, each group of the symmetrically germinated seeds was placed in the seven flasks of inoculation treatments and incubated for 4 h at room temperature on a shaker (180 rpm). After incubation, the inoculated seeds were transplanted in alcohol sterilized plastic pots (1 Kg capacity) filled with 900 g of sterilized sandy-soil mixture. Four seeds were sown in each pot, grown in a greenhouse at 25–30 °C, and irrigated as required without fertilization. Thinning was performed after two weeks of sowing, leaving three homologous plants per pot. The experimental design consisted of triplicate pots for each treatment.

##### Plant Tissue Analyses

After 30 days, the plants were harvested, the root and shoot systems were carefully separated, and the root systems were washed with running tap water to remove soil residuals. The effectiveness of plant growth promotion was assessed by measuring the height and dry weights for both the root and shoot systems. Subsamples of the fresh shoot were used for assessing the plant proteolytic, lipolytic, and amylolytic activities. One gram of a fresh shoot sample was homogenized with 10 mL phosphate buffer pH 6.8 (0.1 M), then diluted to 100 mL in additional phosphate buffer. The homogeneous solution was centrifuged at 2 °C for 30 min at 5000 rpm and the clear supernatant (containing the enzymes) was then transferred to a 100 mL dialysis bag. Dialysis was done using crystalline sucrose at 4 °C for 24 h to obtain a 10× concentrated dialysate. The enzymatic activity of maize plants was assessed using the agar well diffusion method [35,36]. Water agar media supplemented with 1% gelatin, 1% tributyrin, and 1% soluble starch was used for assaying protease, lipase, and amylase, respectively. After incubation for 24 h, a reagent composed of acidic mercuric chloride for protease or 1% iodine for amylase was added. The diameter of the clearing zone around each well was measured to estimate the enzyme activity.

For chemical analysis, a known weight of dried samples was digested and Na^+^ and K^+^ contents were evaluated following the method described by Wolf [37], using a flame photometer (Jenway Flame Photometer, Bibby Scientific Ltd-Stone-Staffs-St15 0SA-UK). P was measured spectrophotometrically according to Cottenie et al. [38] using a Jenway 6305 UV spectrophotometer at a wavelength of 690 nm.

### 2.7. Statistical Analysis

Data were analyzed statistically using SPSS v17 (SPSS Inc., Chicago, IL, USA). One-way analysis of variance (ANOVA) was used to assess the effect of fungal inoculations on PGP properties; antimicrobial activity, P-solubilization ability, ammonia, and IAA production, and the effect of these inoculants on the performance of maize growth. Posterior multiple comparisons were done using Tukey’s range tests at *p* < 0.05. All results are the means of three independent replicates.

## 3. Results and Discussion

### 3.1. Isolation and Identification of Culturable Endophytic Fungi

**Figure 1:**
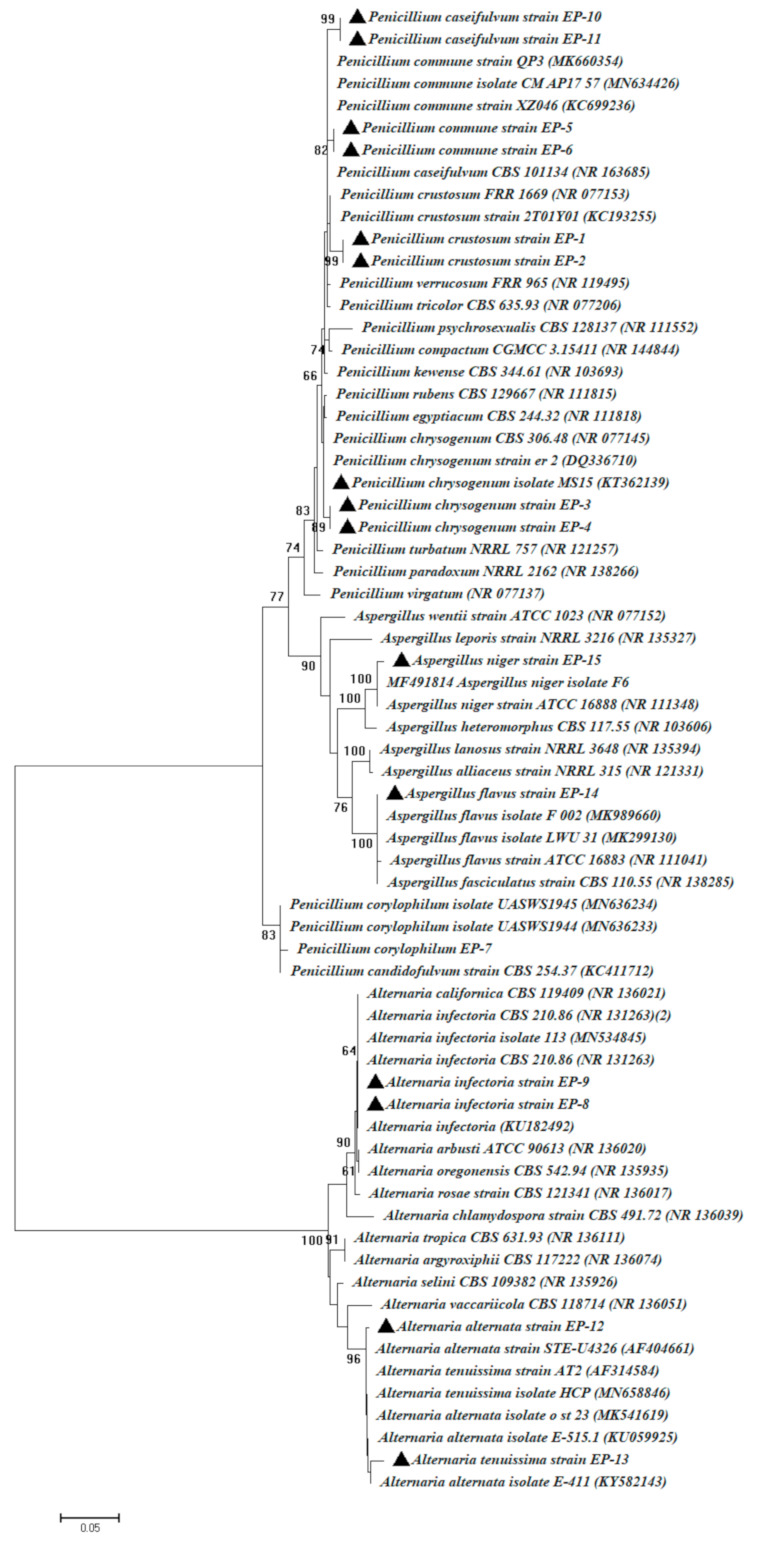
Phylogenetic analysis of ITS sequences of fungal strains with reference sequences retrieved from NCBI (National Center for Biotechnology Information). EP-1–EP-15 refer to the ITS sequences of fungal isolated from *Ephedra pachyclada* plants. The identity of the fungal isolates is available in Table 1. The analysis was implemented in MEGA 6 using the neighbor-joining method.

Endophytic microorganisms contribute to the growth of plants and recovery of plant health by different strategies including control of phytopathogens, secretion of phytohormones such as IAA, cytokinins, and gibberellic acids. Additionally, endophytes can reinforce plant growth by nitrogen fixation, phosphate solubilization, nutrient cycling, and secretion of novel and bioactive metabolites. Secondary metabolites secreted by endophytic microbes have various biotechnological applications [39,40]. In this study, 15 endophytic fungi were isolated from mature leaves of *E. pachyclada*; based on the ITS sequence analysis, fungal isolates were identified as: *Penicillium crustosum* (EP-1 and EP-2), *Penicillium chrysogenum* (EP-3 and EP-4), *Penicillium commune* (EP-5 and EP-6), *Penicillium corylophilum* (EP-7), *Alternaria infectoria* (EP-8 and EP-9), *Penicillium caseifulvum* (EP-10 and EP-11), *Alternaria alternata* (EP-12), *Alternaria tenuissima* (EP-13), *Aspergillus flavus* (EP-14), and *Aspergillus niger* (EP-15). The BLAST analysis of these fungal strains revealed 98–99% identity (Table 1) with ITS sequences of the related species **(**Figure 1**)**. There have been a few reports about endophytic fungi obtained from the *Ephedraceae* family, including *Ephedra nebrodensis* [19], *Ephedra alata* and *Ephedra aphylla* [17,18], and *Ephedra intermedia* [41]. To the best of our knowledge, this the first report about the isolation, characterization, and study of plant growth-promoting activity of endophytic fungi obtained from *Ephedra pachyclada* and applied in vivo to improve the growth performance of maize plants.

### 3.2. Assessment of Endophytic Fungal Strains as Plant Growth-Promoting

#### 3.2.1. Extracellular Enzymatic Activities

The potency of isolated fungal endophytes for extracellular enzymatic production was qualitatively evaluated by the agar plate method. Sixty percent of the isolated fungi exhibited enzymatic activity for all the tested enzymes with varying degrees. Meanwhile, 33% of fungal isolates showed their ability to produce four enzymes out of the tested enzymes.

Results revealed that isolate EP-13 has the maximum pectinase and xylanase activities with clear zones 41.67 ± 1.66 mm and 43.67 ± 0.66 mm, respectively. On the other hand, the highest amylase and gelatinase activities were recorded for the isolate EP-12 with clear zones 39.33 ± 0.66 mm. The isolate EP-11 proved to be a potential cellulase producer with the maximum ability to degrade CMC and exhibit a clear zone 41.67 ± 0.88 mm after adding their reagent (Table 2). Interestingly, all endophytic fungal isolates exhibited catalase enzymatic activity; the catalase enzyme is considered the first line of defense in microbes, which protected them against harmful free radicals which arise from biotic and abiotic stresses, and hence, promote plant growth through an indirect strategy [42]. Regarding biotechnological applications, the hydrolytic enzymes secreted by fungal endophytes are used to improve the industrial handling of proteins and polysaccharides degradation [11]. In the same context, the hydrolytic enzymatic activities such as amylase, CMCase, pectinase, gelatinase, and xylanase are correlated with hyperparasitic activity and help fungi to penetrate plant cells [43]. Additionally, these enzymatic activities enhance induced systematic resistance [44]. Kavamura et al. [45] suggested that hydrolytic enzymes secreted by endophytes can promote plant growth by the suppression of plant diseases caused by soil-borne pathogens.

#### 3.2.2. Antimicrobial Activity

The discovery of new active substances that have antimicrobial activity from endophytic fungi has received attention; moreover, the use of endophytic microbes as bioinoculants should be characterized by the inhibitory effect against different pathogens to protect the plant from diseases, then directly promoting plant growth [46].

Herein, the antimicrobial activity of the 15 endophytic fungal strains was assessed against six pathogens including three Gram-negative bacteria (*Escherichia coli*, ATCC 8739; *Pseudomonas aeruginosa*, ATCC 9027, and *Salmonella typhimurium* ATCC 14028), two Gram-positive bacteria (*Staphylococcus aureus*, ATCC 6538, and *Bacillus subtilis*, ATCC 6633) and one unicellular fungus (*Candida albicans*, ATCC 10231). The ethyl acetate extracts of all isolated fungi demonstrated various inhibitory activity against target microbes using an agar diffusion assay.

The crude extract of three endophytic fungal strains, *Penicillium crustosum* EP-1, *Penicillium commune* EP-6, and *Penicillium corylophilum* EP-7 (20% of isolates), showed broad-spectrum activity as they inhibited the growth of Gram-positive and Gram-negative bacteria with IDZ values between 12 to 20 mm. Moreover, four fungal strains (26.6% of isolates) were identified as *Alternaria infectoria* EP-9, *Penicillium caseifulvum* EP-10, *Alternaria tenuissima* EP-13, and *Aspergillus flavus* EP-14, which showed significant repression of the growth of all tested Gram-negative bacteria. On the other hand, two fungal strains, *Penicillium crustosum* EP-2 and *Alternaria alternata* EP-12 (13.3% of isolates), exhibited antibacterial activity against *Staphylococcus aureus* and *Bacillus subtilis* with IDZ ranging between 12 to 20 mm. However, the remaining six fungal strains (40% of isolates) inhibited the growth of at least one Gram-negative or Gram-positive bacteria. In the same context, crude extract of four fungal isolates EP-6, EP-7, EP-9, and EP-11 exhibit antifungal activity against *C. albicans* with IDZ ranges 21 to 25 mm (Table 3). The higher IDZ values observed on *C. albicans* may be attributed to asymmetrical eukaryotic characteristics between endophytic fungal strains and *C. albicans* [47]. The IDZ values are identified based on inhibition zones where the clear zones are less than 10 mm, which are known as low activity, while IDZ values from 10 to 20 mm are considered of medium activity and IDZ values greater than 20 mm are considered to be highly active [48]. Similarly, Manganyi et al. [49] reported that among 133 endophytic fungi isolated from healthy roots and leaves of medicinal plant *Pelargonium sidoides*, only the crude extract of sixteen fungal strains showed antibacterial activity against *Bacillus cereus, Enterococcus faecium, E. coli*, and *Enterococcus gallinarum*. Moreover, the ethyl acetate extract of five endophytic fungal strains isolated from Nigerian plants Ocimum gratissimum, Newbouldia laevis, and Carica papaya displayed antibacterial activity against Gram-positive and Gram-negative bacteria, while they did not exhibit any antifungal activity against *Candida albicans* and *Aspergillus fumigatus* [50].

#### 3.2.3. Phosphate Solubilization and Ammonia Production

Phosphate solubilization and ammonia production are critical mechanisms for promoting plant growth through endophytic fungi [51]. Phosphorus is one of the macronutrients of which a high amount is required for plant growth promotion. In most cases, phosphorus is present in the soil as insoluble inorganic forms; interestingly, different rhizospheric and endophytic fungal strains have the efficacy to convert it from an unavailable to available source for plant uptake [52]. In this study, the phosphate solubilizing activity for all fungal strains was qualitatively assessed on pikoviskaya media supplemented with tri-calcium phosphate as an inorganic phosphate source. Out of the isolated endophytic fungal strains, only three isolates *Penicillium crustosum* EP-2, *Penicillium chrysogenum* EP-3, and *Aspergillus flavus* EP-14 were able to form clear zones, suggesting a phosphate solubilizing ability. The isolate *Aspergillus flavus* EP-14 recorded the maximum solubilization zone with a clear zone of 13 mm (Table 4). Phosphate solubilizing fungi can be used for increasing crop production and promote plant growth by phosphate availability [53]. On the other hand, ammonia can be indirect to promote plant growth by suppression of plant-pathogen [54]. Additionally, endophytic fungi that producing ammonia can supply plants with a sufficient amount of ammonia required for root and shoot elongations and consequently promote plant growth [55]. Herein, all isolated fungal strains had the ability to produce ammonia with a varying degree after adding Nessler’s reagent to broth media (Table 4). These results are inconsistent with Ripa et al. [9], who reported that, out of 16 endophytic fungal strains associated with the *Triticum aestivum* plant, only six strains had the ability for ammonia production.

#### 3.2.4. Qualitative and Quantitative IAA Productions

**Figure 2:**
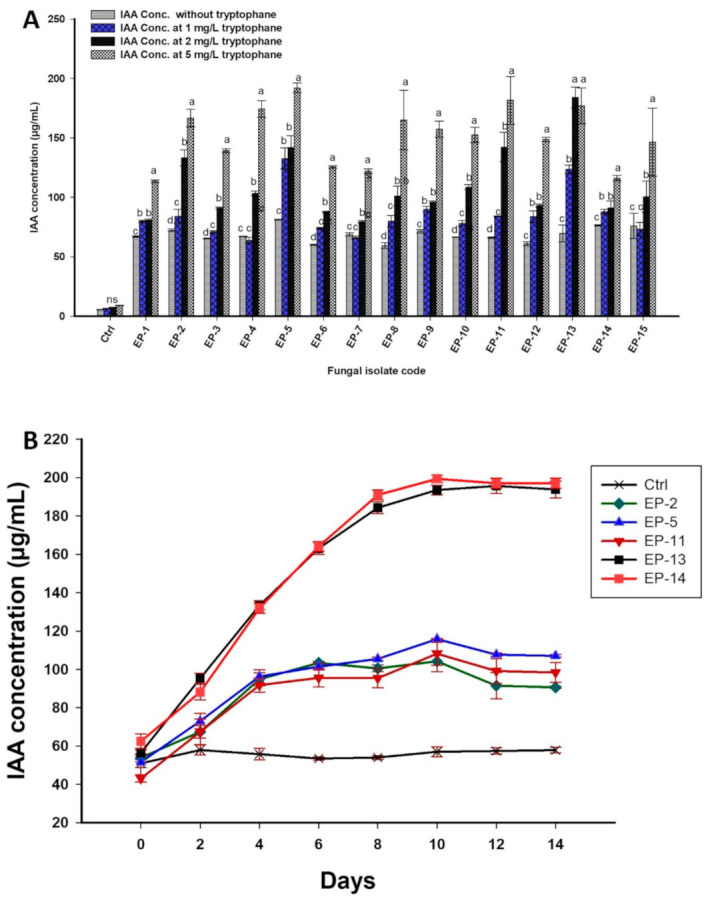
(**A**) Quantitative production of IAA by 15 endophytic fungal strains in the absence/presence of different concentrations of tryptophan (1.0, 2.0, and 5.0 mg/L) after 7 days of incubation. (**B**) IAA production by the most potent five fungal strains in the presence of 5 mg/mL tryptophan and over 14 days. Ctrl, controls without fungal inoculation. Data are statistically different at *p* ≤ 0.05 by Tukey’s test, (*n* = 3); error bars are means ± SE. For each strain, bars with different letters (a, b, c, and d) denote that mean values are significantly different at a significance level of *p* ≤ 0.05. The standard error is less than the size of symbols if no error bars are seen.

The efficiency of fungal endophytes to phytohormones production is a critical factor for promoting plant growth. IAA is the major phytohormones responsible for cell growth, building tissues of xylem and phloem, promoting abscission, and increasing roots growth and elongation, which in turn promote plant growth through nutrient uptake [56,57]. Additionally, IAA contributes to plant-microbes interactions [58]. In this study, all endophytic fungal strains exhibited qualitative IAA production after 14 days of incubation, with and without tryptophan, which was used as a precursor for IAA synthesis. In the absence of tryptophan in growth media, all fungal isolates significantly (*p* ≤ 0.05) produced IAA when compared to control. In this regard, fungal isolates *Penicillium commune* EP-5, *Aspergillus flavus* EP-14, and *Aspergillus niger* EP-15 were able to synthesis high IAA concentrations of 81.3 ± 0.3, 76.4 ± 0.7, and 75.9 ± 10.7 µg mL^−1^, respectively (Figure 2A). On the other hand, *Penicillium commune* EP-5 and *Alternaria tenuissima* EP-13 were highly significant (*p* ≤ 0.001) in IAA production with values 132.8 ± 8.8 and 123.7 ± 3.4 µg mL^−1^, respectively, as compared to other isolates (*p* ≤ 0.01) in the presence of 1 mg mL^-1^ tryptophan. Data analysis showed that in the presence of 2 mg mL^−1^ tryptophan in growth media, fungal isolates EP-13, EP-11, EP-5, and EP-2 significantly (*p* ≤ 0.001) produced higher IAA as compared to other isolates. Fungal isolates EP-5, EP-11, EP-13, EP-4, and EP-2 were high IAA producers (*p* ≤ 0.001), with values 192.1 ± 4.04, 181.6 ± 19.9, 176.9 ± 15, 174.4 ± 7.01, and 166.6 ± 7.3 µg mL^-1^, respectively, in the presence of 5 mg L^−1^ tryptophan as compared to other fungal isolates (Figure 2A). Therefore, endophytic fungal isolates *Penicillium crustosum* EP-2, *Penicillium commune* EP-5, *Penicillium caseifulvum* EP-11, *Alternaria tenuissima* EP-13, and *Aspergillus flavus* EP-14 were selected for quantitative IAA production at different interval times in the presence of 5 mg mL^−1^ tryptophan (Figure 2B). Quantitative data analysis revealed that maximum IAA production (83.2 ± 2.7 and 84.5 ± 4.9 µg mL^−1^) was achieved after 2 days for fungal isolates EP-2 and EP-14, while the maximum productivity for *Penicillium commune* EP-5 and *Alternaria tenuissima* EP-13 were realized after 4 incubation days with values 87.5 ± 11.2 and 168.1 ± 15.9 µg mL^−1^. In the same regard, fungal isolate *Penicillium caseifulvum* EP-11 showed maximum IAA productivity (173.87 ± 32.67 µg mL^−1^) after 10 incubation days. Statistical analysis showed that, after 2 days of incubation, IAA production by endophytic fungal isolates *Aspergillus flavus* EP-14, *Penicillium crustosum* EP-2, and *Penicillium commune* EP-5 were significantly increased (*F*_5,12_ = 12; *p* ≤ 0.001) compared to those recorded by isolates *Penicillium caseifulvum* EP-11 and *Alternaria tenuissima* EP-13. On the other hand, fungal isolate EP-13 has recorded the highest IAA producer as compared to EP-5, EP-11, and EP-14, which were similar IAA producers after 4 incubation days (*F*_5,12_ = 23.7; *p* ≤ 0.001). Moreover, within the incubation time from 6 to 14 days, an analysis of variance showed that the IAA production significantly differed between different fungal isolates (*p* ≤ 0.001). These results are compatible with Fouda et al. [12], who reported that the maximum IAA productivity by *Penicillium chrysogenum* Pc_25, *Sterile hyphae* Sh_26, and *Alternaria alternate* Aa_27 were achieved in the presence of 5 mg mL^−1^ tryptophan in broth media. Ripa et al. [9] reported that 56% of the endophytic fungi isolated from *Triticum aestivum* L. can produce IAA, and the highest productivity was recorded for *Fusarium incarnatum*, with a value of 36.12 ± 0.004 µg mL^−1^. The variation in IAA productions among the same species may be attributed to genetic factors that regulate productivity [59]. These genetic factors are also controlled by various pathways induced by different ways in the same microorganism [60].

### 3.3. Effect of Endophytic Fungal Inoculation on Root Growth

**Figure 3:**
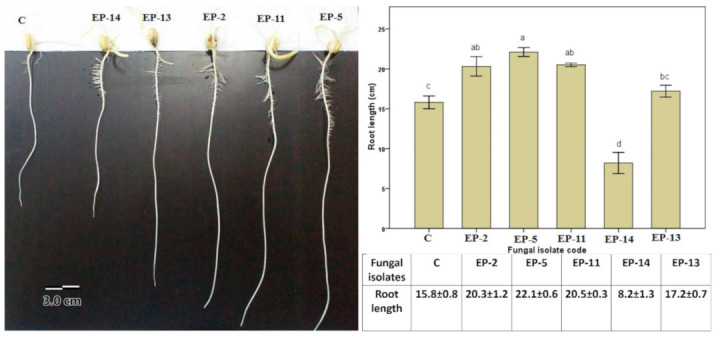
Effect of endophytic fungi as bioinoculant on root length of *Zea mays* L. Error bars are ± SE (*n* = 5). Different letters (a, b, c, and d) on bars denote that mean values are significantly different (*p* ≤ 0.05) by the Tukey LSD test.

The data analysis revealed that the significant enhancement of maize root length was due to bioinculations with endophytic fungal strains (F_5,24_ = 31.86; *p* ≤ 0.001) (Figure 3). The results showed that inoculation with fungal endophytic P*. crustosum* EP-2, *P. caseifulvum* EP-11, and *P. commune* EP-5 significantly increased root length as compared to control non-inoculated and fungal treatments of *A. flavus* EP-14 and *A. tenuissima* EP-13 (*p* ≤ 0.05). However, inoculation by fungal *A. tenuissima* EP-13 and control treatment showed similar effects on root length; indeed, inoculation with fungal *A. flavus* EP-14 significantly reduced root length as compared to other treatments. The significant enhancement of root growth may be attributed to the ability of endophytic fungal strains to produce plant growth-promoting substances such as ammonia, phosphate solubilization, and IAA production. In the same regard, Fouda et al. [12] reported that the ability of endophytic fungi *P. chrysogenum* Pc_25, *Sterile hyphae* Sh_26, and *A. alternate* Aa_27 to increase plant root growth due to plant growth-promoting characteristics.

### 3.4. Greenhouse Experiment

In the current study, fungal endophytes with high efficiency in the production of plant growth-promoting substances were selected to assess their effect on maize growth performance. An analysis of variance revealed that differences in fresh shoot weights of maize plants were significantly (F_6,28_ = 2.82; *p* = 0.028) different between treatments, in the order of EP-2 > EP-11 > mix_F > EP-14 > EP-5 > EP-13 > control **(**Table 5). Additionally, fresh root weights were significantly different due to fungal treatment (F_6,28_ = 2.65; *p* = 0.037). The fresh root weight of maize plants inoculated by Mix_F was higher than those inoculated by fungal EP-11 (Table 5). Neither of the dry weights of shoots (F_6,28_ = 0.98; *p* = 0.454) and roots (F_6,28_ = 0.912; *p* = 0.501) was influenced by fungal treatments; however, the controls tended to show the lowest dry shoot weights. Treatment with fungal EP-11 tended to show the highest dry shoot weights, and fungal EP-2 produced the highest dry root weights even though not significant. Del Carmen Orozco-Mosqueda et al. [61] reported that nutrient transfer efficiency between plant and endophytic microbes are correlated with plant-microbes interaction. Therefore, the variance in weight of shoot and root in plants inoculated with endophytic fungi may be attributed to nutrient availability between plants and microbes.

**Figure 4:**
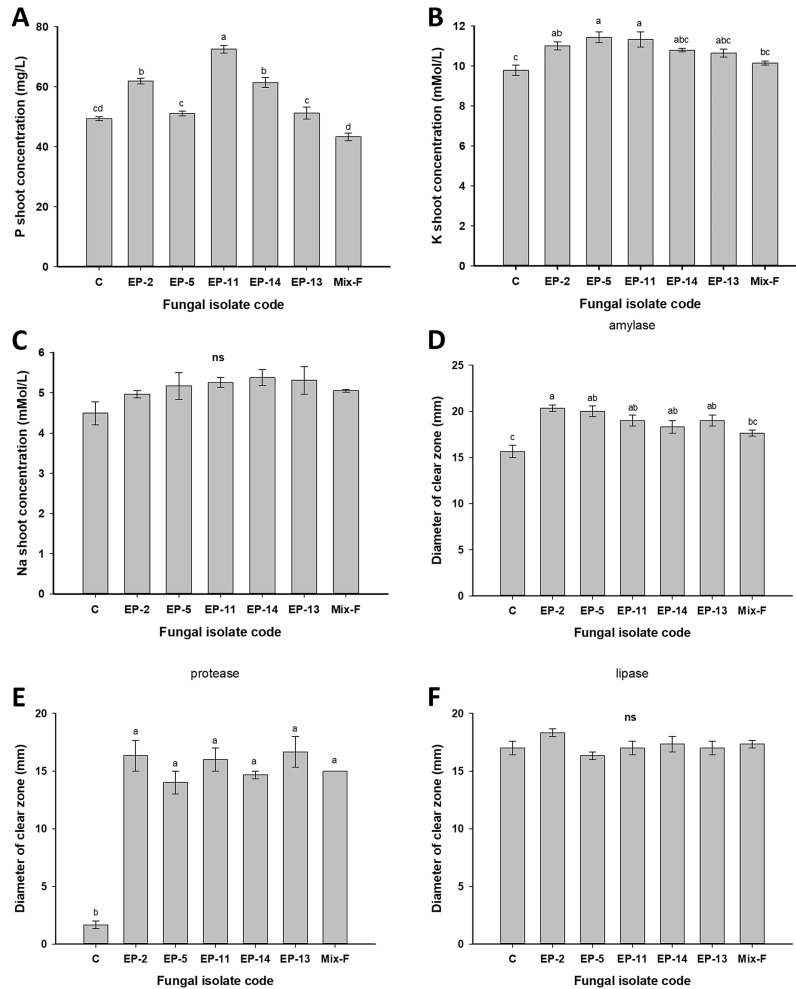
Effect of fungal inoculation on *Zea mays* L. plant growth performance. (**A**) P shoot concentration; (**B**) K shoot concentration; (**C**) Na shoot concentration; (**D**) amylase plant extracellular enzyme activity; (**E**) protease plant extracellular enzyme activity and (**F**) lipase plant extracellular enzyme activity. Mix_F is the consortium between five fungal endophytic strains. Error bars are ± SE (*n* = 3). Different letters on bars denote that mean values are significantly different (*p* ≤ 0.05) by the Tukey LSD test. ns denotes not significant.

Data analysis showed that the shoot P concentration was significantly increased (F_6,14_ = 56.17; *p* ≤ 0.001) in maize plants inoculated with endophytic fungi EP-14, EP-2, and EP-11 than those recorded by EP-13, EP-5, Mix_F, and control (Figure 4A). Moreover, among the tested endophytes, plants inoculated with fungal isolates EP-2, EP-11, and EP-5 significantly showed the maximum shoot K concentration (F_6,14_ = 6.64; *p* = 0.002), whereas plants inoculated with Mix_F or non-inoculated controls exhibited the minimum shoot K concentration (Figure 4B). The results also showed that plants inoculated with different fungal endophytes or plants that were non-inoculated had a similar shoot Na concentration (F_6,14_ = 1.69; *p* = 0.196) (Figure 4C). The main advantages of fungal endophytes are the absorption of the immobile nutrients from the soil, which subsequently convert and transfer these nutrients to plant tissues through various strategies that differ between fungal species [62].

The analysis of variances revealed the significant differences in amylase activities (F_6,14_ = 8.19; *p* = 0.001) of maize plants inoculated with different fungal isolates, in the order of EP-2 > EP-5 > EP-13 > EP-11 > EP-14 > Mix_F > control (Figure 4D). The data analysis showed that the highest protease activities were observed in plants inoculated with fungal isolate EP-13 (F_6,14_ = 33.96; *p* ≤ 0.001), in contrast to non-inoculated control plants, which showed the lowest protease activities (Figure 4E). ANOVA analysis showed variations between different fungal endophytic isolates to hydrolyze lipid substrates (F_6,14_ = 1.43; *p* ≤ 0.269), but these did not reach a significant level (Figure 4F). Regarding the biotechnological view, the endophytic fungi associated with *E. pachyclada* can be used as bioinculants in agricultural fields, not only due to the suppression of pathogens but also due to their plant growth-promoting actions.

Based on in vitro and greenhouse data, it can be concluded that the efficacy of endophytic fungal strain to produce highly valuable active compounds such as hydrolytic enzymes can be used in biotechnological applications. Moreover, the activities of these strains to produce active compounds against pathogenic microbes can be helpful in biomedicine. Interestingly, the efficacy of these strains to phosphate solubilizing, ammonia production, and IAA production confirms the possibility of using these strains in agricultural sectors instead of chemical fertilizers.

## 4. Conclusions

The present study showed that the medicinal plant *E. pachyclada*, which naturally inhabits arid and semi-arid regions, is an ecological niche for diverse endophytic fungi. Therefore, 15 fungal strains were isolated from healthy *E. pachyclada* leaves and identified based on ITS sequence analysis. These endophytes showed varied activities that were plant growth-promoting. Among these activities, the secretion of different extracellular lytic enzymes (pectinase, amylase, cellulase, and catalase) enables them to penetrate tissues. Moreover, the identified fungal strains showed activities against pathogenic different pathogenic microbes which may increase plant resistance to pathogens. Interestingly, the endophytic fungal strains exhibit a variable capacity for phosphate solubilization and produced well-characterized plant-growth regulators (IAA) in the presence/absence of tryptophan. In vivo, five representative endophytic fungi belonging to *P. crustosum* EP-2, *P. commune* EP-5, *P. caseifulvum*, *A. flavus* EP-14, and *A. tenuissima* EP-13, separately and in a consortium (Mix_F), were used as bioinoculants for improving maize plant growth. They stimulated plant growth, increased biomass production, and improved nutrient uptake as compared to uninoculated plants. The obtained data reveal the potentiality of endophytic fungi isolated from the medicinal plant as a source for the synthesis of different bioactive compounds, which can be incorporated into biomedical applications, especially against human pathogenic microbes. Additionally, this work emphasizes the importance of endophytic fungi in agricultural sectors as eco-friendly biofertilizers to improve the plant growth performance or defense, enhance plant production, and improve soil quality and fertility.

## Figures and Tables

**Table 1:** The ITS sequence identification of the endophytic fungal strains from *E. Pachyclada* Boiss.

Fungal Strain Code	GenBank Accession Number	Homolog Sequences	Sequence Identity %	Closest Accession Number
EP-1	MN954764	*Penicillium crustosum*	98.9%	NR077153
			98.5%	NR163685
EP-2	MN954765	*Penicillium crustosum*	98.7%	NR077163
EP-3	MN954766	*Penicillium chrysogenum*	99.3%	NR077145
EP-4	MN954767	*Penicillium chrysogenum*	99.3%	NR077145
EP-5	MN954768	*Penicillium commune*	99.6%	MN634426
			99.6%	MN634422
EP-6	MN954769	*Penicillium commune*	99.6%	MN634426
			99.6%	MN634422
EP-7	MN954770	*Penicillium corylophilum*	99.4%	MN636234
			99.4%	MN636233
EP-8	MN954771	*Alternaria infectoria*	99.8%	KU182492
EP-9	MN954772	*Alternaria infectoria*	99.8%	MN534845
			99.8%	MH205934
			99.8%	MK801346
EP-10	MN954773	*Penicillium caseifulvum*	99.04%	NR163685
EP-11	MN954774	*Penicillium caseifulvum*	99.04%	NR163685
EP-12	MN954775	*Alternaria alternata*	99.6%	KY582143
			99.6%	KX622121
			99.6%	KU059951
EP-13	MN954776	*Alternaria tenuissima*	99.4%	AF314584
EP-14	MN954777	*Aspergillus flavus*	99.6%	NR111041
EP-15	MN954778	*Aspergillus niger*	99.4%	LC195003

**Table 2:** Enzymatic activities of endophytic fungi obtained from *E. pachyclada.*

Fungal Isolate Code	The Diameter of Clear Zones (mm)					
	Amylase	CMCase	Gelatinase	Pectinase	Xylanase	Catalase
Control	0	0	0	0	0	0
EP-1	20.7 ± 0.7	36.3 ± 0.7	30.0 ± 2.9	37.0 ± 1.0	23.7 ± 0.7	+
EP-2	32.7 ± 1.5	37.0 ± 1.0	0	35.3 ± 0.3	26.3 ± 0.7	+
EP-3	8.7 ± 0.7	40.7 ± 0.7	0	34.0 ± 3.1	0	+
EP-4	0	37.0 ± 1.0	26.3 ± 0.9	35.3 ± 1.5	28.3 ± 0.7	+
EP-5	0	28.7 ± 0.7	37.0 ± 1.0	27.3 ± 0.7	25.7 ± 1.2	+
EP-6	34.3 ± 2.3	39.3 ± 1.3	0	32.7 ± 1.5	40.7 ± 0.7	+
EP-7	38.7 ± 0.7	39.3 ± 0.3	22.0 ± 1.2	38.3 ± 0.9	0	+
EP-8	31.7 ± 1.7	38.7 ± 0.7	36.7 ± 0.7	37.7 ± 1.5	41.7 ± 0.9	+
EP-9	23.3 ± 1.7	39.3 ± 0.7	28.3 ± 1.7	40.0 ± 1.2	32.7 ± 0.7	+
EP-10	29.3 ± 2.3	41.3 ± 0.7	39.03 ± 0.7	40.7 ± 0.7	32.7 ± 0.7	+
EP-11	32.3 ± 1.5	41.7 ± 0.9	33.7 ± 1.9	41.0 ± 2.1	39.3 ± 0.7	+
EP-12	39.3 ± 0.7	37.3 ± 1.8	39.3 ± 0.7	41.0 ± 1.0	42.7 ± 1.3	+
EP-13	32.7 ± 1.5	36.0 ± 1.0	38.0 ± 1.2	41.7 ± 1.7	43.7 ± 0.7	+
EP-14	27.7 ± 1.5	38.7 ± 0.7	33.7 ± 1.9	35.3 ± 0.3	25.7 ± 1.3	+
EP-15	37.7 ± 1.5	39.3 ± 1.8	33.3 ± 1.7	39.3 ± 0.7	43.7 ± 0.9	+

0, no activities; +, oxygen bubbles are formed after adding H_2_O_2_ reagent.

**Table 3:** Antimicrobial activities of fungal endophytes isolated from *E. pachyclada.*

Fungal Isolate Code	Diameter of Clear Zones (mm)					
	*P. aeruginosa*	*S. typhimurium*	*E. coli*	*S. aureus*	*B. subtilis*	*C. albicans*
Control	0	0	0	0	0	0
EP-1	16.0 ± 0.8	14.2 ± 0.7	14.3 ± 0.9	13.7 ± 0.7	15.0 ± 0.6	0
EP-2	0	0	0	20.3 ± 0.8	15.0 ± 0.7	0
EP-3	15.0 ± 0.6	0	12.3 ± 0.7	14.7 ± 1.03	13.0 ± 0.9	0
EP-4	18.0 ± 0.7	0	12.3 ± 0.8	0	14.3 ± 0.6	0
EP-5	13.0 ± 0.9	15.3 ± 0.9	0	0	30.0 ± 1.7	0
EP-6	16.0 ± 0.4	12 ± 0.6	20.3 ± 0.6	16.3 ± 0.7	16.7 ± 0.9	25.3 ± 1.3
EP-7	13.0 ± 1.3	14.3 ± 0.5	15.3 ± 0.8	20.3 ± 0.9	14.7 ± 0.9	21.7 ± 1.7
EP-8	14.0 ± 0.9	0	15 ± 0.9	0	0	0
EP-9	18.0 ± 0.7	11.7 ± 0.7	15.3 ± 0.7	0	0	23.3 ± 3.3
EP-10	15.0 ± 0.8	13.7 ± 0.6	14.3 ± 0.6	0	0	0
EP-11	20.3 ± 0.6	14.0 ± 0.9	0	0	0	21.3 ± 0.7
EP-12	15.0 ± 0.9	15.6 ± 1.8	0	15.7 ± 0.7	12.0 ± 0.5	0
EP-13	14.0 ± 0.3	12 ± 0.6	13.3 ± 0.8	16 ± 0.8	0	0
EP-14	22.6 ± 0.8	25.4 ± 1.4	20 ± 0.8	0	20.3 ± 0.9	0
EP-15	16.0 ± 1.1	16.3 ± 0.7	0	0	0	0

**Table 4:** Phosphate solubilizing activities and ammonia production of endophytic fungal strains isolated from *Ephedra Pachyclada.*

Fungal Isolate Code	Phosphate Solubilizing Activity (Diameter of A Clear Zone (mm)	Ammonia Production
Control	0 ^a^	0
EP-1	0 ^a^	+++
EP-2	11.3 ± 0.7 ^b^	+++
EP-3	10.3 ± 0.3 ^bc^	+++
EP-4	0 ^a^	+++
EP-5	0 ^a^	+++
EP-6	0 ^a^	+++
EP-7	0 ^a^	++
EP-8	0 ^a^	+++
EP-9	0 ^a^	+++
EP-10	0 ^a^	++
EP-11	0 ^a^	++
EP-12	0 ^a^	+++
EP-13	0 ^a^	+++
EP-14	13.0 ± 0.6 ^d^	+++
EP-15	0 ^a^	++

In a column, values are the means ± SE (*n* = 3) followed by different letters (a,b,c, and d) which are significantly different (*p* ≤ 0.05) by Tukey LSD (Least Significant Difference) test. ++, yellow color (i.e., highly ammonia production); +++, deep yellow to brownish color (i.e., maximum ammonia production).

**Table 5:** Effect of fungal inoculation on root and shoot of the *Zea mays* L. plant.

Fungal Isolate Code	Fresh Weight (mg)		Dry Weight (mg)	
	Shoot	Root	Shoot	Root
C	597 ± 21.9 ^b^	986.6 ± 37.5 ^ab^	84.4 ± 5.02 ^ab^	309.3 ± 16.9 ^a^
EP-2	793 ± 68.4 ^a^	1112.2 ± 52.7 ^ab^	88.7 ± 5.3 ^ab^	333 ± 22.1 ^a^
EP-5	637.2 ± 34.1 ^ab^	1104.6 ± 82.4 ^ab^	97.9 ± 6.9 ^ab^	278.6 ± 19.3 ^a^
EP-11	694.6 ± 37.2 ^ab^	794.2 ± 56.8 ^b^	100.04 ± 7.2 ^b^	328.2 ± 14.7 ^a^
EP-14	659.2 ± 27 ^ab^	1103.8 ± 124.7 ^ab^	88.9 ± 4.1 ^ab^	298.9 ± 22.3 ^a^
EP-13	614.4 ± 37.1 ^b^	1038.2 ± 67.9 ^ab^	88.2 ± 2.02 ^ab^	317.2 ± 20.2 ^a^
Mix_F	676.6 ± 25.6 ^ab^	1177.8 ± 87.6 ^a^	99.9 ± 11.1 ^a^	293.9 ± 25.1 ^a^

In the columns, values are the means ± SE (*n* = 5) followed by different letters which are significantly different (*p* ≤ 0.05) by Tukey LSD test. Mix_F is the consortium between five fungal endophytic strains.

## Data Availability

The data presented in this study are available on request from the corresponding author.
